# A Metabolomics Coupled With Chemometrics Strategy to Filter Combinatorial Discriminatory Quality Markers of Crude and Salt-Fired *Eucommiae Cortex*

**DOI:** 10.3389/fphar.2020.00838

**Published:** 2020-06-17

**Authors:** Jiading Guo, Jin Li, Xuejing Yang, Hui Wang, Jun He, Erwei Liu, Xiumei Gao, Yan-xu Chang

**Affiliations:** ^1^Tianjin State Key Laboratory of Modern Chinese Medicine, Tianjin University of Traditional Chinese Medicine, Tianjin, China; ^2^Tianjin Key Laboratory of Phytochemistry and Pharmaceutical Analysis, Tianjin University of Traditional Chinese Medicine, Tianjin, China; ^3^School of Pharmacy, Harbin University of Commerce, Harbin, China

**Keywords:** *Eucommiae Cortex*, ultra-high performance liquid chromatography coupled with mass spectrometry, metabolomics, chemometrics, combinatorial discriminatory quality markers

## Abstract

*Eucommiae Cortex* is commonly used for treating various diseases in a form of the crude and salt-fired products. Generally, it is empirical to distinguish the difference between two types of *Eucommiae Cortex*. The metabolomics coupled with chemometrics strategy was proposed to filter the combinatorial discriminatory quality markers for precise distinction and further quality control of the crude and salt-fired *Eucommiae Cortex*. The metabolomics data of multiple batches of *Eucommiae Cortex* samples was obtained by ultra-high performance liquid chromatography coupled with mass spectrometry (UHPLC-MS). Orthogonal partial least-squares discriminant analysis was utilized to filter candidate markers for characterizing the obvious difference of the crude and salt-fired *Eucommiae Cortex*. The accuracy of combinatorial markers was validated by random forest and partial least squares regression. Finally, eleven combinatorial discriminatory quality markers from 67 identified compounds were rapidly screened, identified, and determined for distinguishing the difference between crude and salt-fired *Eucommiae Cortex*. It was demonstrated that UHPLC-MS based metabolomics with chemometrics was a powerful strategy to screen the combinatorial discriminatory quality markers for distinguishing the crude and salt-fired *Eucommiae Cortex* and to provide the reference for precise quality control of *Eucommiae Cortex*.

## Introduction

*Eucommiae Cortex*, also named Duzhong in China, is the dry bark of *Eucommia ulmoides* Oliv. tree and one of the oldest traditional chinese herbal medicines (Zhao et al., 2015). It has been listed as one of the “Middle grade” medicines in Sheng Nong’s herbal classic since two thousand years ago (Cronquist and Takhtadzhian, 1981). It is used clinically to treat a variety of diseases such as osteoporosis, rheumatoid arthritis, hypertension, and menopause syndrome (He et al., 2014). The active ingredients mainly included lignans, iridoids, phenolics, and so on. These ingredients have a wide range of pharmacological activities such as antihypertensive, anti-aging, antioxidant, antimutagenic, and anti-inflammatory activities (Li et al., 2014; Zhu and Sun, 2018).

Traditional Chinese Herbs (TCHs) have been widely used to treat various diseases over thousand years and its global demand is also increasing year after year. Generally, most of Chinese herbs should be prepared in several special processing ways such as stir-frying, steaming, boiling, stewing, and so on (Wang F. et al., 2017). This may directly change the content of some certain compounds, possibly affecting the pharmacological activities of TCHs (Wu et al., 2018). As officially recorded in Chinese pharmacopeia (2015 edition), both the crude and salt-fired *Eucommiae Cortex* commonly used to treat the disease in clinic. Moreover, salt-fired herb medicines are more preferred to act on the “kidney channel” and further improve kidney and liver function according to the Chinese medicine traditional processing theory. Modern research showed that the content and absorption behavior of active compounds would be obviously changed when the *Eucommiae Cortex* was subject to the salt-fired processing (Lu et al., 2018). Importantly, it needs to be clearly stated whether the crude or the salt-fired *Eucommiae Cortex* is used for Chinese medicine prescriptions. For example, the salt-fired *Eucommiae Cortex* was explicitly prescribed to be used for Yougui Wan, Tianma Wan and Qing’e Wan. Compared with the chemical drugs, herbal medicines are the mixtures of multicompounds, which would bring a huge challenge for prescribing the appropriate compounds for quality evaluation. The chemical marker of quality control (QC) of TCHs is commonly one or a few compound (Li et al., 2019). In Chinese Pharmacopeia (2015 edition), the quality standard of *Eucommiae Cortex* is that the content of pinoresinol di-*o*-glucopyranoside is not less than 0.1%. However, it may be not specific and practical due to a lack of definitive standard used for distinguishing two types of *Eucommiae Cortex* products. Therefore, it is necessary to discover the effective quality markers for distinguishing the crude and salt-fired *Eucommiae Cortex*.

Recently, metabolomic technology has become an important and valuable tool in the life sciences. It has been extended to a variety of research areas such as biomarker discovery, disease diagnosis, and quality evaluation of TCHs (Mao et al., 2017; Aszyk et al., 2018). Fortunately, metabolomic methods greatly contribute to discoveries of difference markers that represent the change in the biological environment caused by the special disturbances. Liquid chromatography-mass spectrometry (LC-MS) method plays an important role in the acquisition of metabolomic dataset and identification of metabolites depending on its high separation capacity and sensitivity (Zhou et al., 2012). Chemometrics is quite versatile due to the perfect combination of mathematics, statistics, and computer science (Ziegel, 2004). It provides many good algorithms to mine and retrieve more valuable chemical information from natural products (Kumar et al., 2014). Among the algorithms, random forest (RF) and partial least squares regression (PLSR) are commonly regarded as the effective tool in classification and accuracy prediction for multivariate data (Xia et al., 2017; Wang et al., 2019). Thus, the use of metabolomics in combination with chemometrics might exhibit the unique advantage for the analysis the discrimination between crude *Eucommiae Cortex* and its processed product.

In this work, an LC-MS based metabolomics coupled with chemometrics strategy was proposed to screen the combinatorial discriminatory quality markers (CdQMs) for distinction of crude and salt-fired *Eucommiae Cortex*. Firstly, a total of the 38 different batches of the crude and salt-fired *Eucommiae Cortex* were subjected to ultra-high performance liquid chromatography coupled with quadrupole time-of-flight mass spectrometry (UHPLC-Q-TOF/MS) analysis for acquisition of the whole chemical profile. Secondly, the CdQMs were stepwise filtered from massive metabolomics data by a series of approaches of chemometrics analysis. To be specific, the filtering process was followed by the several rules: 1) the markers could well distinguish the crude and salt-fired *Eucommiae Cortex*; 2) the markers had high accuracies; 3) the markers were easy to access commercially and quantify. At last, the content of the CdQMs in the crude and salt-fired *Eucommiae Cortex* were analyzed by UHPLC–PDA (photodiode array detector) method and the effectiveness of CdQMs were further validated by discriminant analysis. The LC-MS metabolomics coupled with chemometrics strategy was successfully used to screen the CdQMs for distinguishing the crude and salt-fired *Eucommiae Cortex*.

## Materials and Methods

### Plant Material

A total of 54 batches of crude and salt-fired *Eucommiae Cortex* were used for this study. Among the, the 27 batches of crude samples (C1-C19 and VC1-VC8) and 19 batches of salt-fired samples (S1-S19) were purchased from different drugstores in Tianjin and Hebei province of China. Moreover, according to Chinese Pharmacopeia (2015 edition), we processed eight batches of salt-fired samples (VS1-VS8) using the crude samples (VC1-VC8). All samples were authenticated as *Eucommia ulmoides* Oliver. by Prof. Lin Ma (Tianjin University of Traditional Chinese Medicine). The voucher specimens of *Eucommia ulmoides* Oliv., such as QFNU QFNU0018228, QFNU QFNU0018229, SYS SYS00189991, WUK 0060451, etc., were deposited in Chinese Virtual Herbarium (http://www.cvh.ac.cn/), and corresponding herbarium codes, for example, QFNU, SYS, WUK, etc., were searchable in the NYBG Steere Herbarium (http://sweetgum.nybg.org/science/ih/?_ga=2.40299874.1384373005.1557930148-1052084957.1548409239).

### Chemicals and Reagents

HPLC grade acetonitrile, methanol, and formic acid were obtained from Fisher Scientific (Pittsburg, PA, USA) and Anaqua™ Chemicals Supply (Wilmington, DE, USA), respectively. Deionized water was purified by Milli-Q academic ultra-pure water system (Millipore, Milford, MA, USA). Standard substances such as geniposidic acid, neochlorogenic acid, chlorogenic acid, caffeic acid, geniposide, genipin, pinoresinol di-*o*-glucopyranoside, syringaresinol di-*o*-glucopyranoside, isochlorogenic acid A, pinoresinol *o*-glucopyranoside, and isochlorogenic acid C were purchased from Chengdu Desite Bio-Technology Co., Ltd (Chengdu, China). The purity of all standard substances was more than 98%.

### Preparation of Sample and Standard Substance Solution

#### Preparation of Sample Solution

The samples were powered and passed through 80 mesh sieves. The powder (0.400 g) was accurately weighed and was then extracted by ultrasonic method (40 kHz, 1,200 W) for 20 min at room temperature (28°C) with 50% methanol-water (10 ml). All the sample solution was passed through a 0.22-µm filter membrane and was stored at 4^◦^C for subsequent experiments.

#### Preparation of Standard Substance Stock Solution

Eleven standard substances (geniposidic acid, neochlorogenic acid, chlorogenic acid, caffeic acid, geniposide, genipin, pinoresinol di-*o*-glucopyranoside, syringaresinol di-*o*-glucopyranoside, isochlorogenic acid A, pinoresinol *o*-glucopyranoside, and isochlorogenic acid C) were accurately weighed and respectively dissolved with methanol solvent. The separate standard solutions were then mixed as stock solution for plotting standard curves through stepwise dilution.

### UHPLC-Q-TOF/MS Acquisition Analysis

UHPLC-Q-TOF/MS system was composed of Agilent 1290 UHPLC instrument (Agilent Technologies, Waldbronn, Germany) and Agilent 6520 Q-TOF mass spectrometer (Agilent Corporation, Santa Clara, CA, USA). The mass spectra data was acquired in the negative electrospray ion (ESI) mode. The chromatographic peaks were separated on an ACQUITY UPLC BEH C_18_ Column (2.1 × 150 mm, 1.7 µm, Waters) at a flow rate of 0.3 ml/min. The temperature of column was at room temperature (28°C). Mobile phase consisted of 0.1% formic acid–water (A) and acetonitrile (B). The gradient elution program was set as: 0–2.5 min, 5%–10% B; 2.5–7 min, 10%–13% B; 7–10 min, 13%–15% B; 10–11 min, 15%–18% B; 11–15 min, 18%–30% B; 15–17 min, 30%–45% B; 17–22 min, 45%–95% B; 22–27 min, 95%–5% B. The post run time was 5 min. The injection was 5 µl. The related Q-TOF/MS parameters were listed as follows: drying gas, N_2_; gas flow rate, 11 L/min; drying gas temperature, 330^◦^C; nebulizer gas pressure, 40 psig; capillary voltage, 3500 V; fragmentor voltage, 120 V; skimmer voltage, 65 V; octopole RF, 750 V; collision energy (CE), 20 and 30 V. The scan range of mass spectra was m/z 100 – 1,500.

### UHPLC-PDA Analysis

The quantitative analysis was carried out by the Waters Acquity UHPLC instrument (Waters Corp., Milford, MA, USA) using with PDA. The chromatography column, mobile phase and flow rate setting were as same as UPLC-Q-TOF/MS method. The column temperature was 40°C. The gradient elution program was set as: 0–2.5 min, 5%–10% B; 2.5–5.5 min, 10%–11% B; 5.5–6 min, 11%–12% B; 6–10 min, 12%–15% B; 10–13 min, 15%–17% B; 13–14 min, 17%–25% B; 14–15 min, 25%–5% B. The post run was 3 min. The optimal absorbed wavelengths were respectively 240 nm for geniposidic acid, geniposide, genipin, and pinoresinol di-*o*-glucopyranoside; 227 nm for syringaresinol di-*o*-glucopyranoside; and 327 nm for neochlorogenic acid, chlorogenic acid, caffeic acid, isochlorogenic acid A, and isochlorogenic acid C. In order to make the quantitative analysis more convenient, the multiwavelengths switch method was employed and was set as follows: 1.20–2.15 min, 240–327 nm; 2.15–4.50 min, 327–240 nm; 4.50–7.15 min, 240–227 nm; 7.15–10 min, 227–327; 10–12.24 min, 327–227 nm; 12.24–13.00 min, 227–327 nm. The injection volume was 3 µl.

### Qualitative Analysis Method

Six types of compounds in *Eucommiae Cortex* have been summarized on the basis of the literatures, including lignans, phenylpropanoids, iridoids, phenolic acids, and others. The chemical formula and name of all the compounds collected were imported into an excel file and saved as the.csv form. Then the in-house compounds library of *Eucommiae Cortex* had been completed and was used to quickly found out the compounds of interest from the massive raw MS^2^ data by the function “find by formula” on the Agilent MassHunter Qualitative Workstation Analysis B.07.00 (Agilent Technologies Inc., Santa Clara, CA, USA). At last, the in-depth identification was performed by matching real MS, MS^2^ data from the EIC (extraction ion chromatography) with the related information in the literatures, especially, the characteristic ions and fragment pattern.

### Method Validation

#### UHPLC-Q-TOF/MS Acquisition Method Validation

The precision, repeatability, and stability were investigated to validate the applicability of UHPLC-Q-TOF/MS method by using the QC samples. All sample solutions were mixed in a certain volume to prepare for the QC sample. The six independent QC samples were subject to UHPLC-Q-TOF/MS analysis within one day and three continuous days for evaluating the precision. The same QC sample was injected six times to assess the repeatability of acquisition method. The stability was conducted by analyzing response intensity of the target analytes in the QC samples at 0, 2, 4, 6, 8, 12, and 24 h. All the above validation results were presented as the relative standard deviation (RSD).

#### UHPLC-PDA Quantitative Method Validation

The mixed standard stock solution was stepwise diluted into the different working concentrations required by each calibration curve. Calibration curves required was plotted with the peak area as X-axis and the concentrations of target compounds as Y-axis, respectively. The mixed standard solution containing 11 analytes was gradually diluted into the concentrations where the ratio of signal to noise (*S/N*) was detected as 3 and 10, respectively. These mixed standard solutions were then used to evaluate the limits of detection (LOD) and limits of quantification (LOQ). The precision and accuracy of intra- and interday were analyzed by calculating the RSD values of the mixed standard solutions with three different concentrations (low, medium, and high). The repeatability of UHPLC-PDA quantitative method was evaluated by extraction and analysis of the target compounds in six independent samples. The sample solutions were repeatedly injected six times to explore stability at 0, 2, 4, 6, 8, 10, 12, and 24 h in room temperature (28°C), respectively. The recovery experiment was conducted by adding the certain quantity of 11 standards mixture to the samples and the results were assessed by recovery rate (%).

### Data Analysis

Firstly, all the raw data acquired in (-)-ESI mode were introduced to the R software (R Foundation for Statistical Computing, Vienna, Austria) where all mz values detected would be normalized. Secondly, a mass of above metabolomics data was used for the orthogonal partial least-squares discriminant analysis (OPLS-DA) by the Simca-P (version 14.1, Umetrics, Umea, Sweden) in order to initially filter the candidate compounds. Thirdly, the accuracy of CdQMs was validated by PLSR and RF algorithms on Matlab R2015B (Mathworks, Natick, USA). At last, the discriminant function was used to evaluate the applicability of filtered CdQM and predict the types of unknown *Eucommiae Cortex* products by SPSS 17.0 (SPSS, Chicago, IL, USA).

## Results and Discussion

### UHPLC-Q-TOF/MS Acquisition Method Validation

The retention times (Rt), mass to charge ratios (m/z), and peak areas of 11 CdQMs were employed to calculate the RSD values, which were regarded as the important assessment indicator of precision, repeatability, and stability. It was acceptable that the RSD values were no more than 5%. The RSD values of intra- and interday precisions were all below 3.46%, which displayed a high accuracy of Rt, m/z, and peak areas of target ions in the process of multiple samples analysis by the UHPLC-Q-TOF/MS method. Moreover, the repeatability with the RSDs ranging from 0.00% to 3.86% showed good consistency of results detected by UHPLC-Q-TOF/MS. Finally, the RSDs indicative of stability were within 0.00%–3.30%, demonstrating that sample solutions was enough stable for qualitative detection in 24 h. In conclusion, all the above results (**Table S1**) indicated that this UHPLC-Q-TOF/MS method was applicable and reliable for acquiring the metabolomics data.

### Compound Identification in Crude *Eucommiae Cortex*

#### Acquisition of Chemical Compounds Information

**Figure 1:**
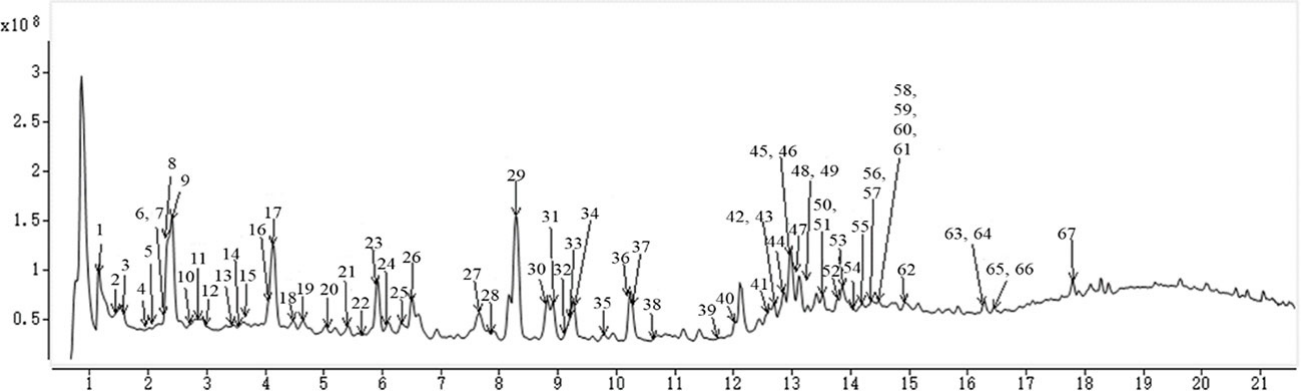
The total ion chromatograms of *Eucommiae Cortex* by ultra-high performance liquid chromatography coupled with mass spectrometry (UHPLC-Q-TOF/MS) in negative ion mode.

The identification of chemical compounds was essential for filtering the candidate markers in the following study. The whole chemical profile of *Eucommiae Cortex* was acquired in the negative ESI mode. In general, the qualitative analysis was time-consuming and labor-intensive due to the massive MS^1^ and MS^2^ data. However, we built the in-house library for the targeted identification, which could rapidly search the known compounds from complex mass spectra data. A total of 72 candidate compounds (**Table S2**) were initially extracted from the MS/MS spectra data. The same compound might hit for several times, whereas the hitting peaks appeared at different retention times. These peaks possibly represented isomers. Therefore, 72 candidate compounds need to be further identified by matching the accuracy MS data (error <5 ppm), key characteristic ions, and chromatographic elution order with that in the literatures to exclude the false positive results. Finally, 67 compounds (**Table 1** and **Figure 1**) in *Eucommiae Cortex* were tentatively identified, containing 31 lignans, 10 iridoids, 10 phenylpropanoids, 6 organic acids, 10 other compounds.

**Table 1:** The identification of constituents of crude *Eucommiae Cortex* extract by UHPLC-Q-TOF/MS in negative ion mode.

Cpd no.	Rt (min)	Formula	[M-H]-	[M+COOH]-	MS/MS(-)	Δppm	Identification		References
1	1.178	C_6_H_8_O_7_	191.0196		111.0076, 129.0178, 154.9993, 173.0085	3.26	Isocitric acid	Others	He et al., 2018; Lei et al., 2018
2	1.45	C_15_H_22_O_9_		391.1231	101.0243, 111.0064, 147.0446, 165.0551, 183.0652	4.29	Aucubin	Iridoids	Allen et al., 2015; He et al., 2018; Jiang et al, 2019
3	1.534	C_16_H_22_O_11_	389.1090		113.0245, 119.0368, 139.0394, 147.0427, 165.0552, 183.0658, 209.0448, 227.0539	0.47	Deacetylasperulosidic acid	Iridoids	He et al., 2018
4	2.077	C_13_H_16_O_9_	315.0714		108.0195, 153.0159	2.39	Protocatechuicacid-4-glucoside	Phenylpropanoids	He et al., 2018
5	2.194	C_15_H_20_O_12_S	423.0598		119.0497, 163.0396, 199.0068, 215.0008, 242.9988, 261.008, 303.1024	1.11	6-(4-Formyl-2,6- dimethoxyphenoxy)-3,4,5- trihydroxytetrahydro-2H-pyran-2-yl) methyl hydrogen sulfate	Others	He et al., 2018
6	2.212	C_14_H_18_O_9_	329.0873		123.0443, 152.011, 167.0342	1.53	2-Glucopyranosyloxy-5- hydroxyphenyl acetic acid	Organic acids	He et al., 2018
7	2.28	C_8_H_8_O_4_	167.0341		108.02303, 123.0413, 152.0104	0.80	Vanillic acid	Organic acids	Lei et al., 2018
8	2.348	C_16_H_22_O_10_	373.1125		101.0242, 123.0447, 147.0431, 149.0602, 167.0704, 193.0497, 211.0606	3.97	Geniposidic acid	Iridoids	Allen et al., 2015; He et al., 2018; Jiang et al., 2019
9	2.534	C_7_H_6_O_4_	153.0186		109.0293	4.78	3,4-Dihydroxy benzoic acid	Organic acids	He et al., 2018; Jia et al., 2019
10	2.753	C_15_H_20_O_10_	359.0981		123.0087, 138.032, 153.05331, 166.9983, 182.0214, 197.0444	0.75	4-Glucopyranosyloxy-3,5- dimethoxy benzoic acid	Organic acids	He et al., 2018; Jia et al., 2019
11	2.874	C_16_H_18_O_9_	353.0883		135.0451, 161.025, 173.0453, 179.0341, 191.0557	-1.40	Neochlorogenic acid	Phenylpropanoids	Allen et al., 2015; He et al., 2018
12	3.008	C_15_H_24_O_10_	363.1281		101.0246, 105.0191, 123.0445, 147.0295	4.31	Harpagide	Iridoids	He et al., 2018
13	3.484	C_13_H_24_O_9_	323.1336		101.0235, 113.0237, 119.0344, 131.0352, 143.0386,	3.63	Periplobiose	Others	He et al., 2018; Jia et al., 2019
14	3.568	C_7_H_6_O_3_	137.0241		108.0218, 109.0282, 119.0153, 136.0158	2.30	3-Hydroxybenzoic acid	Others	He et al., 2018; Jia et al., 2019
15	3.702	C_22_H_28_O_14_	515.1398		179.0351, 191.0555, 323.0786	1.61	cis 5-*o*-(3' -*o*-caffeoyl glucosyl) quinic acid	Phenylpropanoids	He et al., 2018; Jia et al., 2019
16	4.109	C_32_H_44_O_17_		745.2532	179.0706, 195.0657, 327.1237, 345.1349, 357.1306, 375.1444, 537.1926	4.07	Olivil 4' ,4”-di-*o*- glucopyranoside	Lignans	He et al., 2018; Jiang et al., 2019
17	4.16	C_16_H_18_O_9_	353.0883		135.0446, 155.0341, 161.0239, 173.0448, 179.0344, 191.0559	-1.40	Chlorogenic acid	Phenylpropanoids	Allen et al., 2015; He et al., 2018; Jia et al., 2019
18	4.492	C_16_H_18_O_9_	353.0876		135.0440, 155.0351, 161.0225, 173.0444, 179.0340, 191.0554	0.58	Cryptochlorogenic acid	Phenylpropanoids	Allen et al., 2015; He et al., 2018
19	4.636	C_9_H_8_O_4_	179.0355		109.0308, 117.0338, 134.0364, 135.0446	-2.88	Caffeic acid	Phenylpropanoids	Zhang et al., 2016; He et al., 2018
20	5.132	C_17_H_22_O_10_	385.1136		101.0239, 123.0465, 177.0550	1.09	4-[3-Glucopyranosyloxy-2- hydroxyphenyl]-3-methyl-4- oxobutanoic acid	Organic acids	He et al., 2018
21	5.465	C_18_H_26_O_10_		447.1501	101.0242, 111.0083, 134.0326, 149.0487, 161.0453, 233.0655, 251.0941, 269.1011	1.99	4-[2-(Xylopyranosyloxy)ethyl] phenylxylopyranoside	Others	He et al., 2018
22	5.742	C_16_H_18_O_8_	337.0933		161.0239, 163.0398, 191.0555	-1.21	3-p-coumaroylquinic acid	Phenylpropanoids	He et al., 2018
23	5.929	C_17_H_24_O_10_		433.1341	101.0244, 105.0323, 123.0450, 147.0401, 207.0657, 225.0768	-2.70	Geniposide	Iridoids	He et al., 2018; Jia et al., 2019
24	6.07	C_26_H_34_O_12_	537.1956		151.0352, 297.1106, 312.1036, 327.1236, 345.1367, 357.1324	3.99	Olivil 4' -*o*-glucopyranoside	Lignans	He et al., 2018; Jiang et al., 2019
25	6.552	C_32_H_42_O_17_	697.2336		373.1291, 535.1812	1.90	l-Hydroxypinoresinol di-*o*- glucopyranoside	Lignans	He et al., 2018
26	6.62	C_33_H_44_O_19_	743.2380		343.1179, 373.1286, 535.1886	3.23	Naringin DHC 4-*o*- β -d- glucopyranoside	Others	He et al., 2018; Jia et al., 2019
27	7.62	C_11_H_14_O_5_	225.0767		101.0244, 105.0347, 119.0500, 123.0445, 147.0444, 207.0663	0.65	Genipin	Iridoids	Allen et al., 2015; Zhang et al., 2016; He et al., 2018
28	7.907	C_26_H_34_O_12_	537.1956		151.0354, 195.0659, 297.1106, 327.1200, 375.1403	3.99	Olivil 4”-*o*-glucopyranoside	Lignans	He et al., 2018
29	8.162	C_32_H_42_O_16_	681.2373		136.0165, 151.0395, 175.076, 327.1279, 342.1108, 357.1350, 519.1824	3.97	Pinoresinol di-*o*- glucopyranoside	Lignans	Brenes et al., 2000; Feng et al., 2007
30	8.705	C_32_H_42_O_16_	681.2376		179.0536, 339.1248, 01.1789, 219.1864	3.97	Dehydrodiconiferyl 4,γ ‘-*o*- glucopyranoside	Lignans	He et al., 2018
31	8.974	C_17_H_20_O_9_	367.1028		134.0366, 173.0422, 191.0551	1.78	5-*o*-feruloylquinic acid	Phenylpropanoids	He et al., 2018; Jia et al., 2019
32	9.112	C_23_H_26_O_13_	509.1295		123.0442, 153.0181, 182.0212, 197.0451, 211.0608, 297.0624, 311.0764	1.11	4,8,9,10-tetrahydroxy-3,6,7-trimethoxy-2-anthryl-glucopyranoside	Organic acids	He et al., 2018; Jia et al., 2019
33	9.196	C_33_H_46_O_18_	729.2584		165.0553, 491.1922, 503.1920, 521.2033	3.75	3-[4-(2-[4-Glucopyranosyloxy- 3-methoxyphenyl)-2−hydroxy- 1-(hydroxymethyl) ethoxy]- 3,5−dimethoxyphenyl]-2- propen-1-ylglucopyranoside	Others	He et al., 2018
34	9.315	C_33_H_44_O_17_	711.2487		387.1447, 549.1960	2.63	Medioresinol di-*o*- glucopyranoside	Lignans	He et al., 2018
35	9.738	C_26_H_32_O_12_	535.1800		181.0499, 269.0827, 298.082, 313.1096, 325.1005, 343.1183, 358.1068	3.92	l-Hydroxypinoresinol 4'-*o*- glucopyranoside	Lignans	He et al., 2018; Jiang et al., 2019
36	10.196	C_26_H_32_O_12_	535.1800		151.0419, 181.0494, 298.0839, 313.1062, 343.1171, 373.1294	3.92	l-Hydroxypinoresinol 4”-*o*- glucopyranoside	Lignans	He et al., 2018; Jiang et al., 2019
37	10.281	C_34_H_46_O_18_	741.2593		166.0294, 181.0491, 357.1347, 371.1440, 402.1312, 417.1552	2.48	Syringaresinol di-*o*- glucopyranoside	Lignans	Chai et al., 2012; He et al., 2018
38	10.671	C_10_H1_8_O_5_	217.1078		123.0758, 127.0724, 137.0953, 155.1075, 171.1015, 199.0947	1.59	Epieucommiol	Iridoids	He et al., 2018; Jiang et al., 2019; Jia et al., 2019
39	11.838	C_23_H_26_O_13_	509.1292		123.0445, 152.0115, 167.0340, 183.0297, 197.0455, 327.0710	1.69	4-{[6-*o*-(4-hydroxy-3,5- dimethoxybenzoyl)- glucopyranosyl]oxy}-3- methoxybenzoic acid	Others	He et al., 2018
40	12.094	C_25_H_31_NO_11_	520.1811		101.0248, 147.0446	2.56	Eucomoside B	Iridoids	Allen et al., 2015; He et al., 2018
41	12.705	C_25_H_24_O_12_	515.1189		135.0444, 155.0355, 161.0245, 173.0450, 179.0346, 191.0552, 335.0780, 353.0865	1.16	Isochlorogenic acid A	Phenylpropanoids	He et al., 2018; Jia et al., 2019
42	12.774	C_20_H_22_O_7_	373.1291		150.0317, 162.0331, 165.0543, 177.0525, 180.0418	0.47	Erythro-guaiacylglycerol-β - conifery aldehyde ether	Lignans	He et al., 2018
43	12.79	C_43_H_56_O_21_	907.3220		165.0550, 195.0678, 357.1308, 387.1445, 535.1994, 565.2034, 745.2721, 861.2972	2.35	Hedyotol C di-*o*- glucopyranoside	Lignans	He et al., 2018; Jiang et al., 2019
44	12.858	C_43_H_54_O_22_	921.3000		341.0882, 417.1579, 759.2532	3.68	Unknown	Lignans	He et al., 2018
45	12.991	C_20_H_22_O_6_	357.1342		136.0155, 175.0727, 297.1164, 311.1266, 327.0943	0.45	Pinoresinol	Lignans	Brenes et al., 2000
46	12.994	C_26_H_32_O_11_	519.1862		136.0160, 151.0396, 175.0754, 297.1131, 311.1287, 357.1348	1.89	Pinoresinol-*o*-glucopyranoside	Lignans	Qi et al., 2019
47	13.059	C_27_H_34_O_12_	549.1978		136.0154, 166.0269, 181.0505, 372.1212, 387.1451	-0.45	Medioresinol 4”-*o*- β-d- glucopyranoside	Lignans	He et al., 2018; Jiang et al., 2019
48	13.113	C_9_H_16_O_4_	187.0972		123.0813, 143.1073, 169.0864	2.03	Eucommiol	Iridoids	He et al., 2018; Jiang et al., 2019; Jia et al., 2019
49	13.333	C_44_H_58_O_22_	937.3316		387.1405, 891.2881	3.30	Glycerol-syringaresinol ether di-glucopyranoside	Lignans	He et al., 2018
50	13.516	C_28_H_36_O_13_	579.2027		151.0031, 166.0262, 181.0499, 402.1316, 417.1558	1.92	Syringaresinol 4'-*o*- glucopyranoside	Lignans	He et al., 2018; Qi et al., 2019
51	13.604	C_27_H_34_O_12_	549.1978		151.0398, 181.0498, 372.1203, 387.1439, 150.0335	-4.09	Eucommin A	Lignans	He et al., 2018; Qi et al., 2019
52	13.859	C_20_H_22_O_7_	373.1278		162.0344, 165.0574	3.95	Threo-guaiacylglyc erol-β conifery aldehyde ether	Lignans	He et al., 2018
53	13.875	C_25_H_24_O_12_	515.1189		135.0442, 155.0344, 161.0224, 173.0448, 179.0342, 191.0557, 335.0774, 353.0874	1.16	Isochlorogenic acid C	Phenylpropanoids	He et al., 2018; Jia et al., 2019
54	14.063	C_42_H_52_O_21_	891.2918		167.0396, 311.0770, 387.1431, 417.1552	1.16	Syringaresinol vanillic acid ether diglucopyranoside	Lignans	He et al., 2018; Jia et al., 2019
55	14.195	C_40_H_48_O_19_	831.2689		167.0379, 311.0753, 343.1194, 519.1846, 669.2187	3.37	Pinoresinol vanillic acid ether	Lignans	He et al., 2018; Jia et al., 2019
56	14.264	C_15_H_26_O_7_	317.1601		163.1155, 181.1225, 199.1346, 207.1019, 225.1139, 243.1235	1.50	diglucopyranoside 2-(5-Hydroxy-2,3-dimethyl-2- cyclopenten-1- yl)ethylglucopyranoside	Others	He et al., 2018
57	14.265	C_41_H_50_O_20_	861.2794		151.0400, 311.0760, 357.1375, 387.1440, 699.2325	3.33	Medioresinol vanillic acid ether diglucopyranoside	Lignans	He et al., 2018; Jia et al., 2019
58	14.334	C_20_H_22_O_7_	373.1295		136.0157, 181.0514, 188.0471, 269.0825, 285.1125, 298.0855, 313.1083, 358.1049	-0.60	1-Hydroxypinoresinol	Lignans	Pi et al., 2016; He et al., 2018; Jiang et al., 2019
59	14.398	C_21_H_24_O_7_		433.1499	166.0253, 181.0472	1.30	Medioresinol	Lignans	He et al.,2018; Jia et al.,2019
60	14.401	C_40_H_48_O_19_	831.2686		167.0336, 311.0764, 343.1177	3.75	Pinoresinol vanillic acid ether di-*o*-glucoside isomer	Lignans	He et al., 2018; Jiang et al., 2019
61	14.604	C_37_H_46_O_16_	745.2690		151.0388, 165.0550, 181.0477, 195.0643, 341.0859, 357.1361, 387.1446, 535.1957, 583.2191	3.09	Glycerol-medioresinol ether 4”- glucopyranoside	Lignans	He et al., 2018; Jiang et al., 2019; Jia et al., 2019
62	15.094	C_37_H_46_O_16_	745.2688		165.0549, 181.0494, 195.0654, 357.1359, 387.1438, 505.1856, 535.1964, 583.2174	-4.51	Glycerol-medioresinol ether 4”'−glucopyranoside	Lignans	He et al.,2018; Jiang et al., 2019; Jia et al., 2019
63	16.3	C_36_H_42_O_16_	729.2393		167.0367, 181.0493, 311.0771, 341.0870, 387.1440, 403.1367, 417.1560	0.38	Syringaresinol vanillic acid ether glucopyranoside	Lignans	He et al., 2018
64	16.383	C_35_H_40_O_15_	699.2292		151.0388, 167.0342, 197.0446, 311.0778, 341.0881, 357.1340, 387.1440	0.35	Medioresinol vanillic acid ether glucopyranoside	Lignans	He et al., 2018; Jiang et al., 2019; Jia et al., 2019
65	16.586	C_34_H_38_O_14_	669.2182		167.0352, 311.0772, 327.1228, 343.1191, 357.1316	1.01	Pinoresinol vanillic acid ether glucopyranoside	Lignans	He et al., 2018; Jia et al., 2019
66	16.638	C_9_H_16_O_3_	171.1023		153.0902	0.35	1-Deoxyeucommiol	Iridoids	He et al., 2018
67	17.873	C_12_H_20_O_4_	227.1281		143.8607, 165.1225	3.43	5,6,7,8-tetrahydro-7-hydroxy-3,3- dimethyl-1H-cyclopenta[1,3]dioxepin-6-ethanol	Others	He et al., 2018

#### Identification of Lignans

Lignans and their derivatives were a main class of secondary metabolites in *Eucommiae Cortex*, and display various bioactivities *in vivo* or *in vitro* (Deyama, 1983; Shi et al., 2013). In this work, 31 lignans have been tentatively characterized, including compounds **16**, **24-25**, **28-30**, **34-37, 42-47, 49-52**, **54**, **55**, **57-65** (Brenes et al., 2000; Guo et al., 2007; Feng et al., 2007; Chai et al., 2012; Pi et al., 2016; He et al., 2018; Jia et al., 2019; Jiang et al., 2019; Qi et al., 2019). The most lignans in *Eucommiae Cortex* are phenylpropanoid dimers with one or two glucose units, which means a few of the MS^2^ fragments followed by the loss of glucose neutral moiety. Moreover, the MS^2^ spectrum of lignans showed several key characteristic ions at m/z 327, 311, 181, and 150, which were mainly attributed to cleavage of the tetrahydrofuran ring and losses of CH_3_, CH_2_O, CO, CH_3_O, and CH_3_OH (Guo et al., 2007; Jiang et al., 2019). Take several compounds for examples to illustrate the qualitative process. The quasi-molecular ion [M-H]^-^ of compound **29** at m/z 681 corresponded to the formula C_32_H_42_O_16_. Its MS^2^ fragmental ions at m/z 519 and m/z 357 were observed due to the loss of 1 and 2 glucose groups, respectively, and MS^2^ ion at m/z 151 was generated by the cleavage of tetrahydrofuran-ring. The compound **29** was thereof identified as pinoresinol di-*o*-glucopyranoside (Brenes et al., 2000; Feng et al., 2007). The parent ion [C_20_H_22_O_6_-H]^-^ of compound **45** at m/z 357 firstly was converted into the characteristic ion at m/z 327 due to the cleavage of tetrahydrofuran-ring. Moreover, another characteristic ion at m/z 311 was also observed owing to the loss of CH_3_ (15 Da) from the ion at m/z 327. Thus, the compound **45** was tentatively identified as pinoresinol (Brenes et al., 2000). As to the compound **46**, its parent ion [C_26_H_32_O_11_-H]^-^ at m/z 591 was lower 162 Da than that of compound **29**. Additionally, it shared the similar characteristic ions to compound **29** at m/z 311, 297. Finally, compound **46** was rapidly identified as pinoresinol-*o*-glucopyranoside (Qi et al., 2019). The compounds **42** and **52** with [M-H]^-^ ion at m/z 373 had another characteristic ion at m/z 165 and further produced ion with m/z 150 by the loss of CH_3_. Compared the real retention behavior with that in the reported literatures (He et al., 2018), compounds **42** and **52** were tentatively identified as erythro-guaiacylglycerol-β-conifery aldehyde ether and threo-guaiacylglycerol-β-conifery aldehyde ether, respectively. Although several lignans such as compounds **25**, **30**, **34**, **43**, **44**, and **49** could not be found based on the characteristic ions, they were also tentatively identified by comparing with the precise parent ions (error below 5 ppm), MS^2^ fragment ions and the retention behavior with the data obtained in literatures (He et al., 2018; Jiang et al., 2019).

#### Identification of Phenylpropanoids

The phenylpropanoids in *Eucommiae Cortex* were divided into the simple phenylpropanoids and polyol phenylpropanoids, that is, caffeoyl quinic acids. In general, the caffeoyl quinic acids were more prone to produce [caffeoyl]^-^ ion peak at m/z 179 or/and [quinine]^-^ ion peak at m/z 191 (Ouyang et al., 2017; He et al., 2018; Jiang et al., 2019). The MS^2^ ion peak at m/z 173 appeared because one molecule H_2_O was separated from the precursor ion at m/z 191 (Özgen et al., 2009; He et al., 2018; Jiang et al., 2019). Thus, the characteristic diagnosis ion at 191, 179, and/or 173 were used for rapid identification of compounds **11**, **15**, **17**, **18**, **22**, **31**, **41**, and **53**. Compounds **11**, **17**, and **18** exhibited the same molecular ion [M-H]^-^ at m/z 353 and also shared the product ions at m/z 161 and 135 attributed to the loss of one molecular H_2_O (18 Da) and CO_2_ (44 Da) from the [caffeoyl]^-^ ion at m/z 179. According to the information reported, compounds **11**, **17**, and **18** were tentatively speculated as neochlorogenic acid, chlorogenic acid, and cryptochlorogenic acid (Allen et al., 2015; He et al., 2018; Jia et al., 2019), respectively. The cleavage pattern of isomers **41** and **53** were basically consistent with chlorogenic acid isomers. Therefore, it was inferred that compounds **41** and **53** were isochlorogenic acid A and C (He et al., 2018; Jia et al., 2019), respectively. In addition, the simple phenylpropanoids (compounds **4** and **19**) in *Eucommiae Cortex* were cleaved in the different way. The [M−H]^-^ ion of compound **19** (caffeic acid) at m/z 179 produced an [M-H-CO_2_]^-^ ion at m/z 135 and an [M-H-CO_2_-H_2_O]^-^ ion at m/z 117. However, the product ion [M-H-Glc]^-^ of compound **4** (protocatechuicacid-4-glucoside) at m/z 153 eliminated the neutral group CO_2_ to yield the ion at m/z 108 (Zhang et al., 2016; He et al., 2018).

#### Identification of Iridoids

A total of 10 iridoids were identified in this work, including compounds **2**, **3**, **8**, **12**, **23**, **27**, **38**, **40**, **48**, and **66** (Özgen et al., 2009; Allen et al., 2015; Pi et al., 2016; He et al., 2018; Hsueh and Tsai, 2018; Jiang et al., 2019). The most iridoid glycosides were inclining to get aglycon ion due to eliminate glucose neutral group (162 Da). For example, compounds **2**, **3**, **8**, and **23** yielded [M-H-Glc]^-^ ion at m/z 183, 227, 211 and 225 (Allen et al., 2015; He et al., 2018; Jia et al., 2019; Jiang et al., 2019). As reported in the literatures (He et al., 2018; Jiang et al., 2019), the characteristic ions of iridoids were at m/z 101, 119 and/or 147. The identified iridoids except for **38 48**, and **66** showed the characteristic diagnosis ions at m/z 101 and m/z 147 (He et al., 2018). The characteristic ion at m/z 101 was indicative of a CH_2_OH group or CH_3_ and OH groups linked to the C-8 position. Another characteristic ion at m/z 147, was the consequence of successive elimination of glycosidic moiety or loss of H_2_O, CO_2_, HCOOH, and HCOOCH_3_ moiety. The compounds **38**, **48**, and **66** displayed (M-H)^-^ ions at m/z 217, 187 and 171 corresponding to chemical formula C_10_H_18_O_5_, C_9_H_16_O_4_, and C_9_H_16_O_3_. Their MS^2^ ions peak at m/z 199, 169, and 153 were yielded by loss of H_2_O from the parent ion. Moreover, the other MS^2^ ions and retention behavior were consistent with that in the reported literatures (He et al., 2018; Jia et al., 2019). Thus, compounds **38**, **48**, and **66** were probably epieucommiol, eucommiol and 1-deoxyeucommiol.

#### Identification of Phenolic Acids

Compounds **6**, **7**, **9**, **10**, **20**, and **32** (**Table 1**) were tentatively identified on the basis of the key ions at m/z 123 and 153 indicative of the core skeleton similar to derivatives of catechol and 3,4-dihydroxy benzoic acid (He et al., 2018; Lei et al., 2018; Jiang et al., 2019; Jia et al., 2019). Moreover, a few of common neutral fragments such as CH_3_, CO_2_, and glucosyl unit were also recognized as important identification features of phenolic acids. For example, the vanillic acid (compounds **7**) lose the methyl radical (.CH_3_) and one molecule CO_2_ to get fragment ions at m/z 152 and m/z 108, respectively (Lei et al., 2018).

#### Identification of Other Compounds

As to other compounds (**1**, **5**, **13**, **14**, **21**, **26**, **33**, **39**, **56**, and **67**), it was impossible to identify compounds base on the key characteristic ions due to the lack of detail information about shared structure. However, the compounds could be tentatively identified by comparing the experimental data with information of literatures, such as precise MS data and fragment ions (He et al., 2018; Jia et al., 2019; Jiang et al., 2019).

#### Metabolomics Data Analysis

**Figure 2:**
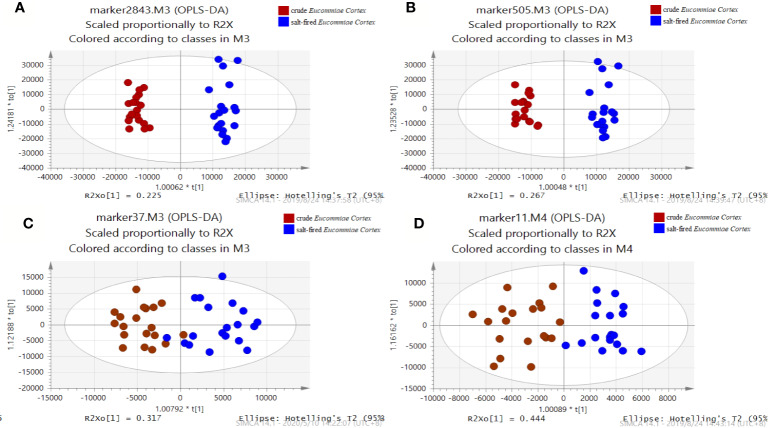
The orthogonal partial least-squares discriminant analysis (OPLS-DA) model for 38 samples of the crude and salt-fired *Eucommiae Cortex* by 2,843 variables **(A)**, 505 variables **(B)**, 37 variables **(C)**, and 11 variables **(D)**, respectively.

Metabolomics analysis has good performance on screening the difference compounds in natural plant samples. Using the R package XCMS, all the raw mass spectra data of C1-C19 and S1-S19 samples, which were acquired from UHPLC-Q-TOF/MS-ESI^-^, was converted into a three-dimensional matrix including information of a mass of variables, such as retention times, m/z values, peak intensities. Then 2,843 variables were generated and were subjected to OPLS-DA analysis on the SIMIC software. OPLS-DA, a supervised multivariate data analysis method, was characterized by difference analysis of inter-groups. The OPLS-DA plot (**Figure 2A**) displayed the obvious separation between crude and salt-fired samples in the presence of 2,843 variables. However, 2,843 variables were not practical for distinction of two types of *Eucommiae Cortex* and even QC assessment. Thus OPLS-DA was further utilized to mine potential and obvious difference compounds based on the value of variable importance parameter (VIP) higher than 1, which was considered to greatly contribute to the separation of clustering. Then a total of 505 candidate compounds were rapidly filtered from 2,843 variables. It was shown (**Figure 2B**) that the crude and salt-fired *Eucommiae Cortex* was well distinguished by the 505 compounds as candidate markers.

### Identification of the Candidate Markers

The 505 candidate markers with the VIP >1 would be explicitly identified on the basis of qualitative study of compounds in *Eucommiae Cortex*. Thirty-seven compounds were rapidly identified from 505 candidate markers according to m/z values and retention time of the significant difference markers. They were respectively compounds **1**, **2**, **3**, **5**, **6**, **8**, **9**, **10**, **11**, **14**, **16**, **17**, **19**, **21**, **23**, **25**, **26**, **27**, **29**, **33**, **34**, **37**, **39**, **40**, **41**, **43**, **44**, **46**, **48**, **49**, **50**, **53**, **60, 63**, **64**, **65**, and **66** (**Table 1**). The other unknown markers would continue to be identified. Moreover, OPLS-DA analysis results (**Figure 2C**) showed that two groups of *Eucommiae Cortex* samples were basically differentiated by the 37 candidate markers. It suggested that the filtered 37 compounds might be potential CdQMs as an alternative to the 505 candidate markers.

### Selection and Verification of the Final CdQMs

Although the range of difference markers was limited to 37 CdQMs in *Eucommiae Cortex* by the analysis of OPLS-DA, it was still considerably difficult to simultaneously achieve the QC and effective distinction of the crude *Eucommiae Cortex* and its salt-fired product. Therefore, it was indeed necessary to further filter the practical CdQMs from the above 37 identified CdQMs. Then the CdQMs would be unambiguously defined according to the following characteristics: easy quantitation, commercial access, and the most importantly, good distinction ability to two types of *Eucommiae Cortex* products. Consequently, eleven compounds (geniposidic acid, neochlorogenic acid, chlorogenic acid, caffeic acid, geniposide, genipin, pinoresinol di-*o*-glucopyranoside, syringaresinol di-*o*-glucopyranoside, isochlorogenic acid A, and isochlorogenic acid C) were roughly selected as potential CdQMs based on the first two characteristics. The OPLS-DA analysis (**Figure 2D**) showed that 11 potential CdQMs could well separate the crude samples and salt-fired samples. However, the accuracy of the selected CdQMs needs to be further validated. Herein, two supervised learning model, the PLSR and RF, were implemented to determine the accuracy of the markers generated *via* each filtering steps. The batches of C1-10 and S1-10 were respectively set as training set of the crude group and the salt-fired group. The remaining batches (C11-19 of crude *Eucommiae Cortex* and S11-19 of salt-fired *Eucommiae Cortex*) were analyzed as testing set. In general, the training set was used to build a model, whereas the testing set was used to verify the established model and provide the accuracies of related variables. Finally, the PLSR and RF algorithms were employed to predict and classify the 38 batches of samples with the 2843, 505, 37, and 11 compounds as variables, respectively. The analysis results of algorithms (**Table 2**) showed the accuracies of 2,843, 505, and 11 variables were all more than 90%, whereas the accuracy of 37 variables were obviously lower than those of others. It demonstrated that the 37 compounds were not optimal candidate markers. Interestingly, the accuracy of 11 variables was equivalent to that of 505 variables, and even close to the accuracy of 2,843 variables. Therefore, the 11 compounds as CdQMs could be fully behalf of the whole compounds in *Eucommiae Cortex* for distinguishing the crude and salt-fired *Eucommiae Cortex* and were used for quality evaluation of two-types of *Eucommiae Cortex* products.

**Table 2:** The accuracies of different variables by PLSR and RF Algorithms.

Algorithms	Different amounts of variables			
	2843	505	38	11
PLSR	100%	100%	66.7%	94.1%
RF	100%	100%	83.3%	94.1%

### UHPLC-PDA Quantitative Method Validation

**Figure 3:**
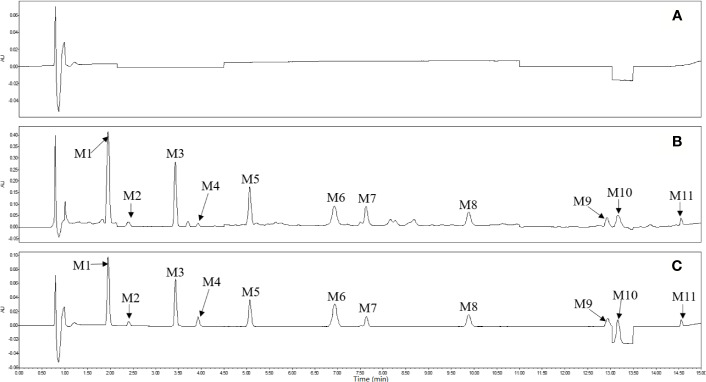
Ultra-high performance liquid chromatography (UHPLC) chromatograms of blank solvent solution **(A)**, sample solution **(B)**, and mixed standard solution **(C)**. M1-11 represented geniposidic acid, neochlorogenic acid, chlorogenic acid, caffeic acid, geniposide, genipin, pinoresinol di-*o*-glucopyranoside, syringaresinol di-*o*-glucopyranoside, isochlorogenic acid A, pinoresinol *o*-glucopyranoside, and isochlorogenic acid C, respectively.

To validate the UHPLC-PDA method, the selectivity, linearity, LOD and LOQ, repeatability, accuracies and precisions, stability, and recoveries should be investigated and the related data was well displayed (**Tables S3** and **S4**). In contrast to the chromatogram of the 11 standard substances and blank solution, obvious interference was not observed in the chromatogram of extract solution (**Figure 3**), indicating that the analytical method had good selectivity for detection of 11 analytes. A total of 11 standard curve lines enabled to accurately determine the concentrations of target components within the analysis range due to the r^2^ values more than 0.9991. The range of LOQs and LODs for 11 CdQMs were from 0.03 to 1.00 µg/ml and 0.01 to 0.3 µg/ml, respectively. The detection method was much stable to determine multisamples due to the RSDs of repeatability below 5%. The intra-day and inter-day accuracies were the range of 88.2%–105% and the RSDs of the corresponding precisions were within 0.10%–4.69%. The results obviously validated the fact that this quantitative method could analyze accurately the samples in several days. The recoveries of this method for the 11 components ranged from 95.0% to 104% (**Table S3**), fully demonstrating the extremely little loss of target compounds in the extraction and sampling process. Overall, this developed UHPLC-PDA method was well fitting for the analysis of the 11 CdQMs in *Eucommiae Cortex* samples.

### Analysis of Different Batches of *Eucommiae Cortex* samples

In order to exclude the influence of origin places on the selection of quality markers, 8 batches of the crude *Eucommiae Cortex* and their salt-fired products from Sichuan Province in China were analyzed using the same OPLS-DA strategy according to the same rules. The same eleven quality markers were also found and filtered. Although VIP values of eleven quality markers (**Table S5**) from the same origin place were different with those of samples from the different origin places, these eleven quality markers could divide these samples into two groups. One is the crude and the other is salt-fired group. This result was basically consistent with the real situation. Thus, the processing could change the chemical contents of 11 CdQMs in crude samples leading to the difference from the salt-fired samples. Based on the above analysis, eleven CdQMs were identified and regarded as the featured markers that could be alternative to the whole chemical compounds profile for differentiation of the crude and the salt-fired *Eucommiae Cortex*.

The validated UHPLC-PDA method was employed to simultaneously determine the content of the 11 CdQMs (geniposidic acid, neochlorogenic acid, chlorogenic acid, caffeic acid, geniposide, genipin, pinoresinol di-*o*-glucopyranoside, syringaresinol di-*o*-glucopyranoside, isochlorogenic acid A, pinoresinol *o*-glucopyranoside, and isochlorogenic acid C) in 54 batches of *Eucommiae Cortex* samples. Among them, the C1-C19 and VC1-VC8 batches were crude *Eucommiae Cortex* and remaining batches (S1-S19 and VS1-VS8) were salt-fired *Eucommiae Cortex*. Base on the average content of each marker (**Table 3**), the contents of nine markers (geniposidic acid, neochlorogenic acid, caffeic acid, geniposide, genipin, pinoresinol di-*o*-glucopyranoside, syringaresinol di-*o*-glucopyranoside, isochlorogenic acid A, and isochlorogenic acid C) were reduced while two markers (chlorogenic acid and pinoresinol *o*-glucopyranoside) were increased after crude *Eucommiae Cortex* samples were salt-fired. The possible reason was relation to the structure transformation of compounds such as oxidation, decomposition, isomerization in the salt-fired process (Wu et al., 2018). Moreover, these CdQMs had a variety of pharmacological activities such as antioxidant, anti-inflammatory, anti-cancer, anti-atherosclerosis, and anti-hypertension (Gao et al., 2015; Li et al., 2015; Liu et al., 2016; Wang J. et al., 2017; Ma et al., 2019; Xia et al., 2019). Thus, content fluctuation of these markers between the crude *Eucommiae Cortex* and its salt-fired product probably lead to change in bioactive effects. Because many factors could affect the content of chemical ingredients in *Eucommiae Cortex*, more in-depth research need to be carried out for clarifying the influence of processing on the multiple chemical ingredients of *Eucommiae Cortex* in the future.

**Table 3:** The average content of 11 CdQMs in different bathes of *Eucommiae Cortex* samples (μg/mg).

Batches	Average content (μg/mg)										
	M1	M2	M3	M4	M5	M6	M7	M8	M9	M10	M11
C	1.65	0.06	0.51	0.05	0.31	0.40	1.81	0.52	0.16	0.32	0.04
S	1.45	0.05	0.54	0.02	0.28	0.08	1.22	0.30	0.13	0.36	0.03

M1-11 represented geniposidic acid, neochlorogenic acid, chlorogenic acid, caffeic acid, geniposide, genipin, pinoresinol di-o-glucopyranoside, syringaresinol di-o- glucopyranoside, isochlorogenic acid A, pinoresinol o-glucopyranoside, and isochlorogenic acid C, respectively; C represented the crude Eucommiae Cortex group

(C1-C19 and VC1-VC8); S represented the salt-fired Eucommiae Cortex group (S1- S19 and VS1-VS8).

### Discriminant Analysis

Discriminant analysis was characterized by predicting classification of the unknown sample. Discriminant analysis was used to determine whether the unknown samples are crude or salt-fired *Eucommiae Cortex. The* crude samples (C1-C19) and salt-fired samples (S1-S19) were labeled as group 1 and group 2 (**Table 4**), respectively. The contents of 11 CdQMs in these samples were used as modeling data to build the unstandardized canonical discriminant model by SPSS software. The samples (VC1-VC8 and VS1-VS8) were selected as testing sample. The discriminant function generated was showed as follows:

**Table 4:** The classification results by discriminant analysis.

Batches	Actual groups	Predictive groups	Discriminant scores
C1	1	1	1.55389
C2	1	1	0.34026
C3	1	1	2.53401
C4	1	2^#^	-0.56361
C5	1	1	1.42251
C6	1	1	1.35273
C7	1	1	2.13122
C8	1	1	1.04150
C9	1	1	0.22947
C10	1	1	0.02528
C11	1	1	2.49143
C12	1	1	1.80828
C13	1	1	3.15104
C14	1	1	0.44475
C15	1	1	0.72497
C16	1	1	2.80181
C17	1	1	0.71904
C18	1	1	1.33373
C19	1	1	2.97135
S1	2	2	-1.73808
S2	2	2	-0.53112
S3	2	2	-1.16221
S4	2	2	-0.71001
S5	2	2	-0.45801
S6	2	2	-1.39650
S7	2	2	-1.94982
S8	2	2	-2.20743
S9	2	2	-1.21746
S10	2	2	-1.99632
S11	2	2	-1.10804
S12	2	2	-2.16652
S13	2	2	-0.07373
S14	2	2	-3.69995
S15	2	2	-0.25776
S16	2	2	-1.15230
S17	2	2	-0.71390
S18	2	2	-1.20901
S19	2	2	-2.76548
VC1	-	1	0.54668
VC2	-	1	1.73195
VC3	-	1	1.58948
VC4	-	1	1.96910
VC5	-	1	1.16438
VC6	-	1	1.64134
VC7	-	1	3.28505
VC8	-	1	0.39950
VS1	-	2	-0.08497
VS2	-	2	-0.46013
VS3	-	2	-0.68333
VS4	-	2	-0.07023
VS5	-	2	-1.09459
VS6	-	2	-3.20495
VS7	-	1^#^	1.26397
VS8	-	2	-0.07440

1, represented the crude Eucommiae Cortex group; 2, represented the salt-fired Eucommiae Cortex group; -, represented the unknown groups; ^#^represented the incorrect classification.

$$\gamma =0.06X_{1}+14.8X_{2}-6.16X_{3}+6.18X_{4}+3.18X_{5}+1.65X_{6}+2.37X_{7}-0.45X_{8}+0.29X_{9}-2.91X_{10}+1.49X_{11}-1.60$$

where X_1_ to X_11_ represented the contents of geniposidic acid, neochlorogenic acid, chlorogenic acid, caffeic acid, geniposide, genipin, pinoresinol di-*o*-glucopyranoside, syringaresinol di-*o*-glucopyranoside, isochlorogenic acid A, pinoresinol *o*-glucopyranoside, and isochlorogenic acid C, respectively; the γ is the discriminant score. The classification accuracies of this model were 97.4% and 78.9% corresponding to originally grouped cases and cross-validation grouped cases, respectively. It demonstrated that the reliability of this discriminant model was acceptable. Discriminant score of each sample was calculated through the discriminant function. Two centroid values of crude *Eucommiae Cortex* and salt-fired *Eucommiae Cortex* group were respectively 1.395 and -1.395. Their sum was the discriminant value. If discriminant score of one sample was higher than 0, it would be classified into the crude *Eucommiae Cortex* group. Otherwise, they would belong to salt-fired *Eucommiae Cortex*. The results of predictive groups (**Table 4**) displayed that most of samples except for C4 in known groups were correctly classified. Only one unclassified sample (VS7) were not correctly predicted. These results demonstrated that simultaneous determination of 11 CdQMs coupled with discriminant analysis could well be used to differentiate the crude and salt-fired *Eucommiae Cortex*.

## Conclusion

The CdQMs were screened for precise quality assessment of crude and salt-fired *Eucommiae Cortex* by LC-MS metabolomics with chemometrics strategy. An in-house component library of *Eucommiae Cortex* was built for rapid search of known compounds, which would make the qualitative analysis efficient and time-saving. Eleven CdQMs including geniposidic acid, neochlorogenic acid, chlorogenic acid, caffeic acid, geniposide, genipin, pinoresinol di-*o*-glucopyranoside, syringaresinol di-*o*-glucopyranoside, isochlorogenic acid A, pinoresinol *o*-glucopyranoside, and isochlorogenic acid C, were screened step by step and could well differentiate the crude and salt-fired *Eucommiae Cortex*. It was concluded that LC-MS metabolomics with chemometrics was a powerful strategy to filter CdQMs for distinguishing the crude and salt-fired *Eucommiae Cortex*. It would provide a reliable reference for the in-depth investigation of difference between the crude and salt-fired *Eucommiae Cortex*.

## Data Availability Statement

All datasets generated for this study are included in the article/**Supplementary Material**.

## Author Contributions

Y-XC and XG designed the experiment. Y-XC and JG analyzed the experimental data. JG, JL, XY, HW, JH, and EL performed the experiment and wrote the manuscript.

## Conflict of Interest

The authors declare that the research was conducted in the absence of any commercial or financial relationships that could be construed as a potential conflict of interest.
