# Supplementary material for: A Metabolomics Coupled With Chemometrics Strategy to Filter Combinatorial Discriminatory Quality Markers of Crude and Salt-Fired Eucommiae Cortex
Source: Front Pharmacol. 2020 Jun 17;11:838. doi: 10.3389/fphar.2020.00838 (PMC7311666; doi:10.3389/fphar.2020.00838)
Supplement: Supplementary file 1 [file DataSheet_1.pdf]

Supplementary Material

A Metabolomics Coupled with Chemometrics Strategy to Filter Combinatorial discriminatory Quality Markers of Crude and Salt-fired *Eucommiae Cortex*

Jiading Guo[1,2], Jin Li[1], Xuejing Yang[1,3], Hui Wang[1,2], Jun He[1,2], Erwei Liu[1,2], Xiumei Gao[1,2], Yan-xu Chang[1,2]*

[1]Tianjin State Key Laboratory of Modern Chinese Medicine, Tianjin University of Traditional Chinese Medicine, Tianjin, 300193, China

[2]Tianjin Key Laboratory of Phytochemistry and Pharmaceutical Analysis, Tianjin University of Traditional Chinese Medicine, Tianjin, China

[3]School of Pharmacy, Harbin University of Commerce, Harbin, Heilongjiang, PR China

* **Correspondence**:

Yan-xu Chang, Tianjin State Key Laboratory of Modern Chinese Medicine, Tianjin University of Traditional Chinese Medicine

Tel.: +86-22-59596163

Fax: +86-22-59596163

E-mail: tcmcyx@tjutcm.edu.cn (Y.-x.Chang)

Table S1. The precision, repeatability, and stability of the qualitative method

compounds	precision			repeatability			stability		
	Rt (%)	m/z (%)	Peak area (%)	Rt (%)	m/z (%)	Peak area (%)	Rt (%)	m/z (%)	Peak area (%)
M1	0.32	0.00	2.09	0.40	0.00	2.63	0.39	0.00	3.30
M2	0.21	0.00	1.20	0.28	0.00	1.00	0.20	0.00	1.65
M3	0.17	0.00	2.22	0.17	0.00	1.44	0.13	0.00	2.27
M4	0.16	0.00	1.18	0.17	0.00	2.07	0.13	0.00	2.22
M5	0.19	0.00	1.13	0.08	0.00	1.61	0.21	0.00	2.14
M6	0.12	0.00	3.46	0.12	0.00	3.86	0.08	0.00	1.06
M7	0.16	0.00	2.05	0.14	0.00	1.98	0.07	0.00	1.98
M8	0.10	0.00	0.74	0.09	0.00	0.71	0.14	0.00	1.42
M9	0.04	0.00	1.09	0.07	0.00	2.04	0.07	0.00	1.31
M10	0.04	0.00	1.02	0.05	0.02	1.22	0.04	0.00	1.37
M11	0.04	0.00	1.40	0.05	0.00	2.22	0.06	0.00	1.29

M1-11 represented geniposidic acid, neochlorogenic acid, chlorogenic acid, caffeic acid, geniposide, genipin, pinoresinol di-*o*-glucopyranoside, syringaresinol di-*o*-glucopyranoside, isochlorogenic acid A, pinoresinol o-glucopyranoside, and isochlorogenic acid C, respectively.

Table S2. The 72 candidate compounds found by formula

Peak no.	Rt (min)	Formula	$[M-H]^-$	$[M+COOH]^-$	Δppm	Identification
1	1.178	$C_6H_8O_7$	191.0196		3.26	isocitric acid
2	1.45	$C_{15}H_{22}O_9$		391.1231	4.29	aucubin
3	1.534	$C_{16}H_{22}O_{11}$	389.1090		0.47	deacetylasperulosidic acid
4	1.754	$C_{15}H_{22}O_{10}$	361.1122		5.03	catalpol
5	2.077	$C_{13}H_{16}O_9$	315.0714		2.39	protocatechuicacid-4-glucoside
6	2.194	$C_{15}H_{20}O_{12}S$	423.0598		1.11	6-(4-formyl-2,6-dimethoxyphenoxy)-3,4,5-trihydroxytetrahydro-2H-pyran-2-yl) methyl hydrogen sulfate
7	2.212	$C_{14}H_{18}O_9$	329.0873		1.53	2-glucopyranosyloxy-5-hydroxyphenyl acetic acid
8	2.228	$C_{10}H_{12}O_4$		241.0720	-1.22	methyl 3-(3,4-dihydroxyphenyl) propanoate
9	2.28	$C_8H_8O_4$	167.0341		0.80	vanillic acid
10	2.348	$C_{16}H_{22}O_{10}$	373.1125		3.97	geniposidic acid
11	2.534	$C_7H_6O_4$	153.0186		4.78	3,4-dihydroxy benzoic acid
12	2.753	$C_{15}H_{20}O_{10}$	359.0981		0.75	4-glucopyranosyloxy-3,5-dimethoxy benzoic acid
13	2.874	$C_{16}H_{18}O_9$	353.0883		-1.40	neochlorogenic acid
14	3.008	$C_{15}H_{24}O_{10}$	363.1281		4.31	harpagide
15	3.484	$C_{13}H_{24}O_9$	323.1336		3.63	periplobiose
16	3.568	$C_7H_6O_3$	137.0241		2.30	3-hydroxybenzoic acid
17	3.652	$C_{11}H_{16}O_6$	243.0862		4.96	5-methoxy-guaiacylglycerol
18	3.652	$C_6H_6O_2$	109.0292		2.75	catechol
19	3.702	$C_{22}H_{28}O_{14}$	515.1398		1.61	isochlorogenic acid A

20	4.109	$C_{32}H_{44}O_{17}$		745.2532	4.07	olivil 4’ ,4”-di-*o*-glucopyranoside
21	4.16	$C_{16}H_{18}O_9$	353.0883		-1.4	neochlorogenic acid
22	4.492	$C_{16}H_{18}O_9$	353.0876		0.58	neochlorogenic acid
23	4.636	$C_9H_8O_4$	179.0355		-2.88	caffeic acid
24	5.132	$C_{17}H_{22}O_{10}$	385.1136		1.09	4-[3-glucopyranosyloxy-2-hydroxyphenyl]-3-methyl-4-oxobutanoic acid
25	5.465	$C_{18}H_{26}O_{10}$		447.1501	1.99	4-[2-(xylopyranosyloxy)ethyl] phenylxylopyranoside
26	5.742	$C_{16}H_{18}O_8$	337.0933		-1.21	3-p-coumaroylquinic acid
27	5.929	C17H24O10		433.1341	-2.70	geniposide
28	6.07	$C_{26}H_{34}O_{12}$	537.1956		3.99	olivil 4’ -*o*-glucopyranoside
29	6.552	$C_{32}H_{42}O_{17}$	697.2336		1.90	l-hydroxypinoresinol di-*o*-glucopyranoside
30	6.62	$C_{33}H_{44}O_{19}$	743.2380		3.23	naringin DHC 4-*o*-β-d-glucopyranoside
31	7.62	$C_{11}H_{14}O_5$	225.0767		0.65	genipin
32	7.907	$C_{26}H_{34}O_{12}$	537.1956		3.99	olivil 4’-*o*-glucopyranoside
33	8.162	$C_{32}H_{42}O_{16}$	681.2373		3.97	pinoresinol di-*o*-glucopyranoside
34	8.450	$C_{10}H_{10}O_4$	193.0497		4.80	methyl 3-phenylacrylate
35	8.705	$C_{32}H_{42}O_{16}$	681.2376		3.97	pinoresinol di-*o*-glucopyranoside
36	8.974	$C_{17}H_{20}O_9$	367.1028		1.78	5-*o*-feruloylquinic acid
37	9.112	$C_{23}H_{26}O_{13}$	509.1295		1.11	4,8,9,10-tetrahydroxy-3,6,7-trimethoxy-2-anthryl−glucopyranoside
38	9.196	$C_{33}H_{46}O_{18}$	729.2584		3.75	3-[4-(2-[4-glucopyranosyloxy-3-methoxyphenyl)-2−hydroxy-1-(hydroxymethyl) ethoxy]-

					3,5−dimethoxyphenyl]-2-propen-1-ylglucopyranoside
39	9.315	$C_{33}H_{44}O_{17}$	711.2487	2.63	medioresinol di-*o*-glucopyranoside
40	9.738	$C_{26}H_{32}O_{12}$	535.1800	3.92	l-hydroxypinoresinol 4’-*o*-glucopyranoside
41	10.196	$C_{26}H_{32}O_{12}$	535.1800	3.92	l-hydroxypinoresinol 4’-*o*-glucopyranoside
42	10.281	$C_{34}H_{46}O_{18}$	741.2593	2.48	syringaresinol di-*o*-glucopyranoside
43	10.671	$C_{10}H_{18}O_{5}$	217.1078	1.59	epieucommiol
44	11.838	$C_{23}H_{26}O_{13}$	509.1292	1.69	4,8,9,10-tetrahydroxy-3,6,7-trimethoxy-2-anthryl-glucopyranoside
45	12.094	$C_{25}H_{31}NO_{11}$	520.1811	2.56	eucomoside B
46	12.705	$C_{25}H_{24}O_{12}$	515.1189	1.16	isochlorogenic acid A
47	12.774	$C_{20}H_{22}O_{7}$	373.1291	0.47	erythro-guaiacylglycerol-β-conifery aldehyde ether
48	12.79	$C_{43}H_{56}O_{21}$	907.3220	2.35	hedyotol C di-*o*-glucopyranoside
49	12.858	$C_{43}H_{54}O_{22}$	921.3000	3.68	unknown
50	12.991	$C_{20}H_{22}O_{6}$	357.1342	0.45	pinoresinol
51	12.994	$C_{26}H_{32}O_{11}$	519.1862	1.89	pinoresinol-*o*-glucopyranoside
52	13.059	$C_{27}H_{34}O_{12}$	549.1978	-0.45	eucommin A
53	13.113	$C_{9}H_{16}O_{4}$	187.0972	2.03	eucommiol
54	13.333	$C_{44}H_{58}O_{22}$	937.3316	3.30	glycerol-syringaresinol ether di-glucopyranoside
55	13.516	$C_{28}H_{36}O_{13}$	579.2027	1.92	syringaresinol 4’ -*o*-glucopyranoside
56	13.604	$C_{27}H_{34}O_{12}$	549.1978	-4.09	eucommin A
57	13.859	$C_{20}H_{22}O_{7}$	373.1278	3.95	erythro-guaiacylglycerol-β-conifery aldehyde ether

58	13.875	$C_{25}H_{24}O_{12}$	515.1189		1.16	isochlorogenic acid A
59	14.063	$C_{42}H_{52}O_{21}$	891.2918		1.16	syringaresinol vanillic acid ether diglucopyranoside
60	14.195	$C_{40}H_{48}O_{19}$	831.2689		3.37	pinoresinol vanillic acid ether diglucopyranoside
61	14.264	$C_{15}H_{26}O_{7}$	317.1601		1.50	2-(5-hydroxy-2,3-dimethyl-2- cyclopenten-1-yl)ethylglucopyranoside
62	14.265	$C_{41}H_{50}O_{20}$	861.2794		3.33	medioresinol vanillic acid ether diglucopyranoside
63	14.334	$C_{20}H_{22}O_{7}$	373.1295		-0.60	erythro-guaiacylglycerol-β -conifery aldehyde ether
64	14.398	$C_{21}H_{24}O_{7}$		433.1499	1.30	medioresinol
65	14.401	$C_{40}H_{48}O_{19}$	831.2686		3.75	pinoresinol vanillic acid ether diglucopyranoside
66	14.604	$C_{37}H_{46}O_{16}$	745.2690		3.09	glycerol-medioresinol ether 4”-glucopyranoside
67	15.094	$C_{37}H_{46}O_{16}$	745.2688		-4.51	glycerol-medioresinol ether 4”-glucopyranoside
68	16.3	$C_{36}H_{42}O_{16}$	729.23937		0.38	syringaresinol vanillic acid ether glucopyranoside
69	16.383	$C_{35}H_{40}O_{15}$	699.2292		0.35	medioresinol vanillic acid ether glucopyranoside
70	16.586	$C_{34}H_{38}O_{14}$	669.2182		1.01	pinoresinol vanillic acid ether glucopyranoside
71	16.638	$C_{9}H_{16}O_{3}$	171.1023		0.35	1-deoxyeucommiol
72	17.873	$C_{12}H_{20}O_{4}$	227.1281		3.43	5,6,7,8-tetrahydro-7-hydroxy-3,3- dimethyl-1Hcyclopenta[1,3]dioxepin-6-ethanol

Table S3. The regressive equations, linear ranges, LODs, LOQs, repeatability, and recoveries of 11 compounds

analytes	regressive equation	r^2	linear range (g/mL)	LOQ (μg/mL)	LOD (μg /mL)	recovery average (%)	recovery RSD (%)	repeatability RSD (%)
M1	y=14242.94x+1974.73	0.9991	1-250	0.4	0.1	95.9±3.6	3.19	1.69
M2	y=25774.93x+58.91	0.9994	0.04-10	0.04	0.01	104±4	3.46	1.87
M3	y=26005.17x-2756.75	0.9995	0.4-100	0.08	0.025	97.4±4.8	4.88	2.00
M4	y=60808.33x-438.04	0.9996	0.04-10	0.03	0.01	96.2±4.1	4.09	3.52
M5	y=18072.88x+855.45	0.9999	0.4-100	0.3	0.01	95.9±3.9	3.92	1.07
M6	y=24973.82x+2116.83	0.9999	0.4-100	0.35	0.15	97.0±4.3	4.38	0.86
M7	y=15837.93x-2843.27	0.9992	1-200	1	0.3	100±3	3.23	2.00
M8	y=15163.16x-629.57	0.9999	0.4-100	1	0.3	103±3	3.21	2.76
M9	y=23407.61x+2085.57	0.9991	0.1-25	0.04	0.01	100±3	3.17	3.35
M10	y=30106.82x-17.81	0.9998	0.4-100	0.4	0.16	94.3±4.1	4.32	1.49
M11	y=25787.85x-199.09	0.9997	0.04-10	0.04	0.16	98.1±4.3	4.56	4.23

M1-11 represented geniposidic acid, neochlorogenic acid, chlorogenic acid, caffeic acid, geniposide, genipin, pinoresinol di-*o*-glucopyranoside, syringaresinol di-*o*-glucopyranoside, isochlorogenic acid A, pinoresinol *o*-glucopyranoside, and isochlorogenic acid C, respectively.

Table S4. The Intra-day and Inter-day accuracy and precision, and stability of 11 markers (n = 6).

Analytes	Concentration (μg/mL)	Inter-day		Intra-day		Stability	
		Accuracy (%)	RSD (%)	Accuracy (%)	RSD (%)	Accuracy (%)	RSD (%)
	2	93.3±2.4	2.30	97.8±2.6	2.60	96.4±2.2	2.16
M1	20	104±0	0.10	104±1	0.72	104±0	0.49
	200	103±0	0.39	103±1	1.54	99.7±1.5	1.52
	0.08	92.9±4.1	3.70	96.4±0.8	0.79	97.1±3.0	2.98
M2	0.8	97.2±1.2	1.17	99.6±2.1	2.06	98.5±1.0	0.97
	8	90.8±1.9	1.70	95.1±3.3	3.07	99.1±1.3	1.30
	0.8	93.1±4.4	3.88	96.9±2.3	2.21	99.8±0.9	0.86
M3	8	91.9±0.8	0.76	94.2±2.2	2.09	91.3±0.6	0.58
	80	92.1±0.7	0.63	96.6±3.3	3.07	97.9±1.3	1.32
	0.08	104±1	1.13	102±1	1.27	101±3	3.32
M4	0.8	96.4±0.6	0.56	97.5±1.1	1.04	96.8±0.7	0.71
	8	98.7±0.4	0.41	101±1	1.35	97.6±3.0	2.83
	0.8	94.8±3.7	3.54	98.0±1.4	1.30	99.1±2.6	2.56
M5	8	96.3±0.4	0.42	99.6±2.5	2.43	100±0	0.48
	80	94.6±0.3	0.27	96.1±1.5	1.48	97.4±2.8	2.60
	0.8	94.8±0.0	3.90	95.0±0.8	0.79	95.5±3.8	3.55
M6	8	94.3±1.9	1.76	96.3±1.1	1.07	93.6±1.2	1.08
	80	92.4±0.2	0.20	94.4±2.1	1.48	92.3±3.9	3.47
	1.6	105±4	4.21	101±1	1.35	107±6	4.18
M7	16	97.1±1.1	1.31	100±2	2.30	103±1	1.01
	160	97.5±0.1	0.11	98.8±1.6	1.58	101±3	2.66
	0.8	90.1±2.7	2.44	95.3±2.5	2.43	94.2±2.0	2.02

M8	8	96.8±0.6	0.59	99.9±2.7	2.05	99.5±0.8	0.82
	80	97.5±0.2	0.19	100±2	1.79	96.9±2.7	2.57
	0.2	88.2±3.5	3.14	92.8±3.2	2.97	88.3±3.0	2.74
M9	2	94.6±1.0	0.95	96.7±1.3	1.22	97.1±1.1	1.07
	20	105±0	0.40	101±3	2.86	103±1	1.29
	0.8	88.6±4.3	3.93	92.7±2.4	2.24	88.9±3.2	2.90
M10	8	95.1±1.0	0.97	97.3±1.4	1.38	97.0±1.5	1.43
	80	96.6±0.2	0.23	98.2±1.8	1.77	96.4±2.8	2.68
	0.08	97.8±4.9	4.69	97.1±2.5	2.40	97.0±2.3	2.19
M11	0.8	94.1±2.0	1.85	95.8±1.6	1.48	96.7±0.6	0.62
	8	93.5±1.4	1.28	97.7±3.1	2.91	96.3±2.8	2.68

M1-11 represented geniposidic acid, neochlorogenic acid, chlorogenic acid, caffeic acid, geniposide, genipin, pinoresinol di-*o*-glucopyranoside, syringaresinol di-*o*-glucopyranoside, isochlorogenic acid A, pinoresinol *o*-glucopyranoside, and isochlorogenic acid C, respectively.

Table S5. The variable importance parameter (VIP) of 11 CdQMs between two types of *Eucommiae Cortex* from same and different origin places by OPLS-DA analysis.

compounds	VIP	
	same origin places	different origin places
genipin	5.41589	6.75122
pinoresinol di-*o*-glucopyranoside	5.05593	6.27523
geniposide	5.57087	5.94117
chlorogenic acid	4.81987	4.97134
caffeic acid	4.06297	2.84501
pinoresinol *o*-glucopyranoside	3.48873	3.26732
isochlorogenic acid C	2.75739	2.55568
geniposidic acid	2.02841	5.41920
neochlorogenic acid	1.87003	1.84273
syringaresinol di-*o*-glucopyranoside	1.77705	2.18564
isochlorogenic acid A	1.60547	1.48496
